# Antibacterial Peptides from Plants: What They Are and How They Probably Work

**DOI:** 10.1155/2011/250349

**Published:** 2011-03-03

**Authors:** Patrícia Barbosa Pelegrini, Rafael Perseghini del Sarto, Osmar Nascimento Silva, Octávio Luiz Franco, Maria Fátima Grossi-de-Sa

**Affiliations:** ^1^Laboratorio de Interação Molecular Planta-Praga I, Embrapa Recursos Genéticos e Biotecnologia, 70770-197 DF, Brazil; ^2^Centro de Analises Proteômicas e Bioquímicas, Pós-Graduação em Ciências Genômicas e Biotecnologia, Universidade Católica de Brasília, 70790-160 DF, Brazil

## Abstract

Plant antibacterial peptides have been isolated from a wide variety of species. They consist of several protein groups with different features, such as the overall charge of the molecule, the content of disulphide bonds, and structural stability under environmental stress. Although the three-dimensional structures of several classes of plant peptides are well determined, the mechanism of action of some of these molecules is still not well defined. However, further studies may provide new evidences for their function on bacterial cell wall. Therefore, this paper focuses on plant peptides that show activity against plant-pathogenic and human-pathogenic bacteria. Furthermore, we describe the folding of several peptides and similarities among their three-dimensional structures. Some hypotheses for their mechanisms of action and attack on the bacterial membrane surface are also proposed.

## 1. Introduction

The first antibacterial peptide isolated from a plant species was a purothionin from wheat flour (*Triticum aestivum*), which has the ability to inhibit the growth of some phytopathogens such as *Pseudomonas solanacearum*, *Xanthomonas campestris *and *Corynebacterium michiganense* [1]. Almost 40 years later, several additional peptides with antibacterial activity have been characterized, represented not only by thionins, now named defensins, but also by other groups of proteins such as cyclotides, glycine-rich proteins, snakins, 2S albumins, and hevein-type proteins [2–5]. Peptides have been isolated from roots, seeds, flowers, stems, and leaves and have demonstrated activities towards phytopathogens, as well as against bacteria pathogenic to humans [2, 3, 6]. Over the years, antibacterial peptides have become an interesting tool for the development of new techniques in the control of crop losses and in the production of novel antibiotics for the treatment of diverse human infections [7, 8].

However, there is still little information about how these peptides affect the pathogen to cause cell death or growth inhibition. The fact that only a few peptide structures have been studied makes it more difficult to clarify the mechanism of action used to cause damage in bacterial cells [9–12]. Furthermore, it is not clear whether plant antibacterial peptides from different protein families present similar sequences, structures, and modes of action, or whether each group behaves in a different manner. Accordingly, this paper intends to explain some of these features of antibacterial peptides from plant sources. Herein, biochemical and structural properties of several peptides from different protein groups demonstrating antibacterial activity are described. Three-dimensional structures already obtained for plant antibacterial peptides are evaluated, and some possible mechanisms of action including cell membrane disruption, growth inhibition, and death are also proposed. Finally, the similarities among many antibacterial peptides are compared, and their most conserved attributes are evaluated.

## 2. What Are Plant Antibacterial Peptides?

Plant antibacterial peptides are of great importance as components of barrier defence and as a constitutive defense response induced upon infection in a wide variety of plants. Based on primary sequence similarity and activity towards bacteria, these peptides are classified into different groups, as described in Table 1.

Members of the defensin family represent the highest number of antibacterial peptides described so far. They are able to inhibit a wide variety of bacterial species, especially the phytopathogenic ones (Table 1). Plant antibacterial peptides are active against bacteria at low concentrations and have been identified in peripheral cell layers of seed and vegetative tissues, in accordance with their function as a primary defense of vulnerable tissues. Most peptides share some general characteristics such as positively charged residues and high cysteine content for the formation of disulphide bonds. However, some antibacterial peptides, such as the peptides isolated from coconut water and the glycine-rich peptide from guava seeds, respectively, have acidic properties and no disulphide bridges. Most peptides have demonstrated activity against a broad range of different bacterial species and are therefore promising candidates for control of bacterial infections.

Many antibacterial peptide families have been isolated from plants. Pp-Thionin, for example, shows activity against *Rhizobium meliloti, Xanthomonas campestris*, *Micrococcus luteus*, and *C. michiganensis* at an IC_50_ < 50 *μ*g/mL. Moreover, Pp-AMP1 and Pp-AMP2 have potent activity against several phytopathogens, including *Erwinia carotovora*, *Agrobacterium radiobacter*, *Agrobacterium rhizogenes*, *Clavibacter michiganensis* and *Curtobacterium flaccumfaciens*, at a concentration varying from 13 to 25 *μ*g/mL. In addition, Circulins A-B and Cyclopsychotride A from the Cyclotides family show antibacterial effects against human pathogens such as *Staphylococcus aureus, Micrococcus luteus, Escherichia coli, Pseudomonas aeruginosa, Proteus vulgaris *and* Klebsiella oxytoca *at micromolar concentrations [7, 12, 18]. Furthermore, hevein-like proteins Ac-AMP1 and Ac-AMP1 cause growth inhibition of *Bacillus megaterium* and *Sarcina lutea* at concentrations of 40 and 250 *μ*g/mL, respectively [34]. The same was observed earlier for peptides from the knottin family such as Mj-AMP1 and Mj-AMP2 [41]. Furthermore, two members of the Impatiens family, Ib-AMP1 and Ib-AMP4, were able to inhibit the growth of *Bacillus subtilis*, *Micrococcus luteus*, *Staphylococcus aureus*, and *Streptococcus faecalis* at very low concentrations [39]. Finally, Lc-LTPs peptides member (Lipid-transfer) inhibits the Gram-negative bacterium *Agrobacterium tumefaciens *[48]. 

## 3. What Are Some Characteristic Aspects of the Structures of Antibacterial Peptides?

Several studies of the structure of individual antibacterial peptides from plant sources have been performed, but only a few reports have made a comparison of their structural similarities and differences [49]. Studies comparing the primary sequences and tertiary structures of antimicrobial peptides from plants show that 33% of them present activity against bacteria, and around 59% are formed by 30 to 50 amino acid residues [50]. Moreover, it was observed that one key characteristic of antibacterial peptides is a high content of cysteine and/or glycine residues [50, 51]. The occurrence of disulphide bridges is also important for enhancing structural stability under diverse stress conditions [49–51]. Additionally, it was observed that the percentage of cysteine residues is higher in peptides with known *β*-sheet structures [51]. This can be compared to an antibacterial peptide belonging to the glycine-rich family and isolated from guava seeds [8]. The structure of this peptide, inferred by molecular modelling studies, consists only of *α*-helices and lacks *β*-sheets. Analysis of the primary sequence revealed no cysteine residues, and thus the peptide is unable to form disulphide bonds [8]. Therefore, evidence suggests that the presence of cysteine residues and *β*-sheet structures may go together, but this does not imply that these are relevant for antibacterial activity. Similar conclusions can be made concerning the presence of glycine residues. Glycine can provide flexibility to peptide structures, but nothing has been confirmed about its possible importance for antimicrobial activity [8]. 

However, there are implications that charged amino acids are relevant for activity against microorganisms. Around 17% of the amino acids in plant antimicrobial peptides are positively or negatively charged. Specifically, arginines and/or lysines comprise more than 70% of all charged residues found in these peptides, while the remaining 30% consists of the negatively charged aspartic acid and glutamic acid [51]. As will be described further in this paper, charged residues seem to have an essential role in activity towards pathogenic bacteria.

Among all antibacterial peptides isolated and characterised from plant sources, only eight have been evaluated in terms of their tertiary structures. Three of these peptides belong to the cyclotide family, and four others are from the defensin group [10–12, 19, 20, 29, 31, 52]. The last peptide is a hevein-like member [34].

Defensins have a typical three-dimensional structure composed of a *α*-helix followed by 2-3 *β*-strands that are stabilised by 3-4 disulphide bridges [19, 20, 29, 53]. This structure can be observed in all members of this group, even among those with different functionality.

Cyclotides are a unique type of peptide in which the N- and C-termini interact to form a cyclic structure [54]. They can be divided into the following two groups: the bracelets, the main feature of which is a three-dimensional structure composed of *α*-helices and strands, and the Mobius, composed mainly of *β*-sheets and turns [28, 54]. Circulin A is a bracelet member, while Circulin B and Kalata B2 are Mobius members. They share conserved cysteine and glycine residues, but their primary sequence identity is not high, especially for comparisons between bracelets and Mobius peptides [54]. Nevertheless, although they present a cyclic conformation, cyclotide tertiary structure is very similar to that of peptides from the defensin family. Earlier studies suggested that cyclotides are mutant variations of defensin genes, leading to structural changes in the peptides over the years [55]. In comparison, the hevein-like member Ac-AMP1 is also composed of a *α*-helix and two *β*-sheets containing 6 cysteine residues that form 3 disulphide bonds [34, 56, 57]. Although it shows characteristics of a chitin binding protein, Ac-AMP's tertiary structure is very similar to that of the defensins, giving it the classification of a hevein-like peptide [34, 56, 57].

**Figure 1:**
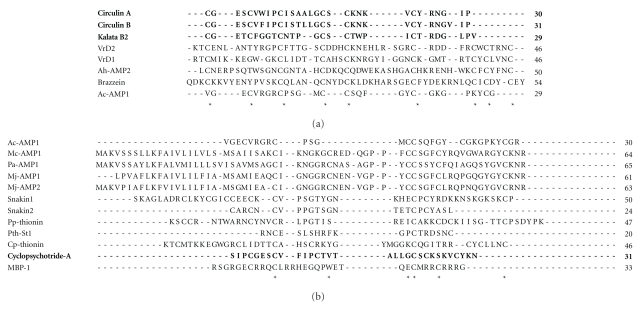
(a) Alignment of antibacterial peptides with defined three-dimensional structure: circulin A (1BH4); circulin B (2ERI); kalata B2 (1PT4); VrD1 (1BK8); VrD2 (2GL1); Ac-AMP2 (1MMC); brazzein (1BRZ). (b) Alignment of several antibacterial peptides from plant sources with sequences available at the Protein Data Bank. Hevein-like: Ac-AMP1 (AAB22103.1); knottin peptides: Mc-AMP1 (081338.1), Pa-AMP1 (P81418.1), Mj-AMP1 (P25403.4), Mj-AMP2 (P25404.2); snakins: snakin1 (AAD01518.1), snakin2 (ABL74292.1); defensins and thionins: pp-Thionin (P07504.1), Pth-St1 (AAB31351.1), and Cp-thionin (P84920.1); cyclotide: cyclopsychotride A (P56872.2); other peptides: MBP-1 (AAB23306.1). Asterisks show conserved cysteine residues. Sequences in bold represent peptides with cyclic three-dimensional conformations, indicating that they do not have N- and C-termini. Therefore, the alignment of cyclotides was based through comparison with the N- and C-termini of the other peptide groups.

An alignment of their primary sequences demonstrates that they present conserved cysteine residues, especially between members of the same family. They also show conservation of glycine residues and a high content of positively charged amino acids (Figures 1(a) and 1(b)).

**Figure 2:**
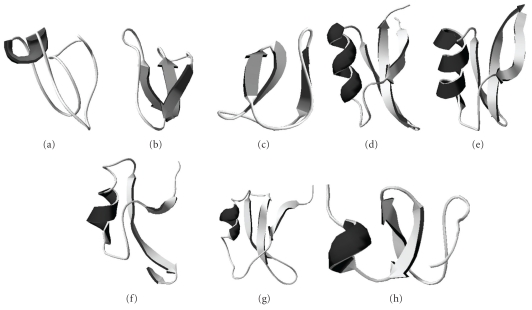
Three-dimensional structure of cyclotides (a) circulin A (PDB: 1BH4); (b) circulin B (PDB: 2ERI); (c) kalata B2 (PDB: 1PT4); defensins (d) VrD1 (PDB: 1TI5); (e) VrD2 (PDB: 2GL1); (f) Ah-AMP1 (PDB: 1BK8); (g) brazzein (1BRZ); and hevein-like (h) Ac-AMP2 (1MMC).

As these peptides belong mainly to 2 families with many features in common, it is possible to see that their three-dimensional conformations are also very similar (Figure 2). When the tertiary structures of all 8 antibacterial peptides for which an NMR structure has been determined are analysed, all are found to be comprised of 1 *α*-helix followed by 2-3 *β*-sheets, with the exception of Circulin B and Kalata B2. In Circulin A, *β*-sheets are replaced by loops (Figure 2(a)). Furthermore, defensins and hevein-like peptides show higher structural similarity to each other than to the cyclotides. Thus, it has not yet become possible to suggest which part of the structure is responsible for antibacterial activity or for conferring specificity against Gram-positive or Gram-negative bacteria.

Among these 8 peptides, Ah-AMP1 is specific towards Gram-positive bacteria, while VrD1 only inhibits Gram-negative bacteria [19, 58]. There is a difference of one *β*-sheet in their structures, but this does not prove that the lack or presence of a *β*-sheet leads to some specific function. Nevertheless, the other 6 peptides display inhibitory activity against both Gram-positive and Gram-negative bacteria, confirming our inability to use only the tertiary conformation to predict peptide functionality. Further studies are necessary to enable the identification of a peptide's function by means of its three-dimensional structure.

## 4. How Do These Peptides Act against Bacteria?

Until now, there have been few reports about the mechanism of action of plant antibacterial peptides. However, these molecules have some important features responsible for antimicrobial activity, including their amphipathic structures and cationic charge at physiological pH [59–62].

The main hypothesis for their mechanism of action involves the ability of AMPs to cause membrane collapse by interacting with lipid molecules on the bacterial cell surface [59–62]. According to this hypothesis, the cationic peptides are attracted electrostatically to negatively charged molecules such as anionic phospholipids, lipopolysaccharides (LPS) (Gram-negative) and teichoic acid (Gram-positive), which are located asymmetrically in the membrane architecture. The positively charged residues can also interact with membrane lipids through specific receptors at the surface of the cell [60, 62]. Consequently, peptide binding to the membrane can activate several pathways that will cause cell death.

However, one general mechanism of action for antibacterial peptides is observed for most peptides. When they reach a threshold concentration, cationic peptides accumulate on the membrane surface in order to direct inner targets for cell lyses. Intrinsic and extrinsic parameters have been reported to influence the threshold peptide concentration. Intrinsic factors include the ability of the peptides to self-assemble and oligomerize, while extrinsic determinants include phospholipid membrane composition, membrane fluidity and head group size; these factors all influence membrane potential, which is critical for determining threshold peptide concentration [62].

**Figure 3:**
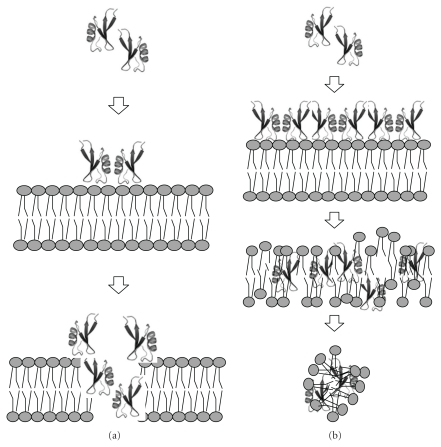
Scheme of the action mechanism for antibacterial peptides. (a) Barrel-stave model; (i) peptides in monomer or oligomer form come close to the membrane target; (ii) positively-charged residues from the peptides interact with the head group of the phospholipids from the membrane; (iii) at a threshold concentration of peptides, the pores are formed. In toroidal model, the major difference is the type of pore formed, where lipids and peptides are overlapped. (b) Carpet model; (i) peptides in monomer or oligomers come close to the membrane target; (ii) hydrophilic regions of peptides are exposed to solvent and hydrophobic regions to membrane; (iii) at threshold concentration of peptides, the permeability of the membrane increases, facilitating pore formation; (iv) membrane disintegration. Adapted from Shai, 2002.

The following 3 processes of pore formation have been reported for plant antibacterial peptides: the barrel-stave mechanism, the toroid pore or wormhole mechanism and the carpet mechanism [59–62]. The barrel-stave mechanism consists of peptide aggregates forming a barrel-ring around an aqueous pore (Figure 3(a)). Peptides interact with the membrane, forcing one thin and hydrophobic portion to bind the phospholipid acyl-chains. After reaching threshold concentration, peptides from the barrel-ring open a pore in the membrane. Their hydrophilic portions comprise the core of the barrel, while the hydrophobic portion interacts with bacterial membrane phospholipids [61, 62].

The toroidal pore or wormhole hypothesis also postulates the formation of pores in a barrel-stave shape. However, in this case, these pores are composed of overlapping peptides and membrane lipids, generating one supramolecular complex. In this structure, the transmembrane pore is formed by peptide and phospholipid head groups. Therefore, the displacement of polar head groups from the peptides induces a positive curvature strain in the membrane by breaching the hydrophobic region [62].

The last mode of action suggested is the carpet mechanism [61, 62]. Initially, peptides in monomeric or oligomeric form bind to the cell surface in an electrostatic manner, covering all the membrane (Figure 3(b)) and giving an appearance of a peptide carpet on the bacterial membrane surface [61]. Consequently, the carpet causes a phospholipid displacement that alters membrane fluidity and/or reduces the barrier properties of membrane. It also leads to membrane disruption and, further, to cell death [62]. Due to the unfavourable energy observed after the membrane bilayer becomes curved, cell rupture and lysis will occur [61]. In this process, the membrane damage occurs in a dispersion-like manner without channel formation [62].

In general, peptides act by formation of membrane pores, resulting in leakage of ions and metabolites, depolarisation and interruption of the respiration process, biopolymer synthesis and cell death [62]. Plant antimicrobial peptides possibly act in the same way as other well-studied antimicrobial peptides [8]. Their folding, an overall positive charge and their amphipathic or amphiphilic nature are essential for antibacterial activity [49]. Furthermore, some studies have shown that antimicrobial peptides from plants can act on intracellular targets [63].

## 5. Concluding Remarks

Antibacterial peptides have been described in many different plant species. They belong to a wide range of protein families, varying from typical antimicrobial members to newly discovered ones. Some peptides show specificity towards Gram-positive or Gram-negative bacteria, but most of them are able to inhibit the activity of both. Therefore, there is at present no way to predict the specificity of any given antimicrobial peptide.

There are few reports describing the tertiary structures of such peptides. However, *in silico *analyses have shown that plant antibacterial peptides present similarities in their three-dimensional structures, although their primary amino acid sequences vary according to the protein family to which they belong. Knowledge of the tertiary structure could yield new insights into the mechanism of action against pathogenic bacteria. Moreover, the description of the mechanism of action for these antibacterial peptides suggests that it may involve a strong interaction with phospholipids from the pathogen's membrane. Parameters such as molecular volume, aggregation ability, and autoassembly onto the membrane surface are essential for activity against bacteria. Indeed, although the mode of action of antibacterial peptides is well-characterised, investigations of the relative importance of specific amino acid residues and their binding with the bacterial cell wall are still in progress.

## Figures and Tables

**Table 1:** Plant antibacterial peptides: physical and antibacterial characteristics.

Peptide	Source	Family	Length	Molecular Mass (Da)	Activity	References
Cn-AMP1	*Cocos nucifera*	—	9	876	Gram+/Gram−	[13]
Cn-AMP2	*Cocos nucifera*	—	11	1266.4	Gram+/Gram−	[13]
Cn-AMP3	*Cocos nucifera*	—	8	905	Gram+/Gram−	[13]
Cy-AMP1	*Cycas revoluta*	—	44	4591.4	Gram+/Gram−	[14]
Cy-AMP2	*Cycas revoluta*	—	44	4577.4	Gram+/Gram−	[14]
Dendrocin	*Dendrocalamus latiflora*	—	35	3863.5	Gram+/Gram−	[15]
Ginkbilobin	*Ginkgo biloba*	—	40	4213.8	Gram+/Gram−	[16]
Lunatusin	*Phaseolus lunatus*	—	20	2178.5	Gram+/Gram−	[17]
Circulin A	*Chassalia parviflora*	Cyclotide	30	3175.8	Gram+/Gram−	[12]
Circulin B	*Chassalia parviflora*	Cyclotide	31	3308	Gram+/Gram−	[7]
Cyclopsychotride A	*Psychotria longipes*	Cyclotide	31	3255	Gram+/Gram−	[18]
Kalata B2	*Oldenlandia affinis*	Cyclotide	29	2979.4	Gram+	[19]
Ah-AMP1	*Aesculus hippocastanum*	Defensin	50		Gram+	[20]
Cp-DefensinII	*Vigna unguiculata*	Defensin	46	5242.3	Gram+/Gram−	[21]
Fabatin-1	*Vicia faba*	Defensin	47	5229.2	Gram+/Gram−	[22]
Fabatin-2	*Vicia faba*	Defensin	47	5206.2	Gram+/Gram−	[22]
Pp-AMP1	*Phyllostachys pubescens*	Defensin	44	4697.4	Gram+/Gram−	[23]
Pp-AMP2	*Phyllostachys pubescens*	Defensin	45	4919.8	Gram+/Gram−	[23]
Pp-Defensin	*Pyrularia pubera*	Defensin	47	5288.2	Gram+/Gram−	[24]
Pth-St1	*Solanum tuberosum*	Defensin	19	2207.4	Gram+/Gram−	[25]
So-D1	*Spinacia oleracea*	Defensin	21	2296.6	Gram+/Gram−	[26]
So-D2	*Spinacia oleracea*	Defensin	52	5803.8	Gram+/Gram−	[26]
So-D3	*Spinacia oleracea*	Defensin	25	2778.3	Gram+/Gram−	[26]
So-D4	*Spinacia oleracea*	Defensin	23	2623.2	Gram+/Gram−	[26]
So-D5	*Spinacia oleracea*	Defensin	24	2737.3	Gram+/Gram−	[26]
So-D6	*Spinacia oleracea*	Defensin	24	2552.9	Gram+/Gram−	[26]
So-D7	*Spinacia oleracea*	Defensin	38	4230.7	Gram+/Gram−	[26]
Tu-AMP 2	*Tulipa gesneriana*	Defensin	20	2259.6	Gram+/Gram−	[27]
Tu-AMP-1	*Tulipa gesneriana*	Defensin	46	4992.9	Gram+/Gram−	[27]
VaD1	*Vigna angularis*	Defensin	45	5009	Gram+/Gram−	[28]
VrD1	*Vigna radiata*	Defensin	46	5140.88	Gram−	[11]
VrD2	*Vigna radiata*	Defensin	47	5503.2	Gram+/Gram−	[29]
White cloud bean defensin	*Phaseolus vulgaris*	Defensin	47	5472.2	Gram+/Gram−	[30]
Brazzein	*Pentadiplandra brazzeana*	Defensin	54	6498.4	Gram+/Gram−	[31]
Sesquin	*Vigna sesquipedalis*	Defensin-like	10	1157.3	Gram+/Gram−	[32]
Coconut antifungal Peptide	*Cocos nucifera*	Glutamic acid-rich	10	1308.3	Gram+/Gram−	[33]
Pg-AMP1	*Psidium guajava*	Glycine-rich	55	6029.4	Gram−	[8]
Ac-AMP1	*Amaranthus caudatus*	Hevein-like	29	3033.6	Gram+	[34]
Ac-AMP2	*Amaranthus caudatus*	Hevein-like	30	3189.8	Gram+	[34]
Ee-CBP	*Euonymus europaeus*	Hevein-like	45	5019.6	Gram+	[35]
Fa-AMP1	*Fagopyrum esculentum*	Hevein-like	40	3887.4	Gram+/Gram−	[36]
Fa-AMP2	*Fagopyrum esculentum*	Hevein-like		3972.5	Gram+/Gram−	[36]
Pn-AMP1	*Pharbitis nil*	Hevein-like	41	4325.9	Gram+/Gram−	[37]
Pn-AMP2	*Pharbitis nil*	Hevein-like	40	4238.8	Gram+/Gram−	[37]
WjAMP1	*Eutrema wasabi*	Hevein-like	40	4094.5	Gram+/Gram−	[38]
Ib-AMP1	*Impatiens balsamina*	Impatiens	20	2558	Gram+/Gram−	[39]
Ib-AMP4	*Impatiens balsamina*	Impatiens	20	2549	Gram+	[39]
Mc-AMP1	*Mesembryanthemum crystallinum*	Knottin	38	4306.59	Gram+	[40]
Mj-AMP1	*Mirabilis jalapa*	Knottin	37	4000.5	Gram+	[41]
Mj-AMP2	*Mirabilis jalapa*	Knottin	36	3893.4	Gram+	[41]
Pa-AMP1	*Phytolacca americana*	Knottin	38	3935.5	Gram+	[42]
Pa-AMP2	*Phytolacca americana*	Knottin	37	3837.5	Gram+	[42]
MBP-1	*Zea mays*	MBP-1	33	4130.7	Gram+/Gram−	[43]
Shepherin I	*Capsella bursa-pastoris*	Shepherin	28	2362.3	Gram+/Gram−	[44]
Shepherin II	*Capsella bursa-pastoris*	Shepherin	38	3259.2	Gram+/Gram−	[44]
Snakin-1	*Solanum tuberosum*	Snakins	63	6934.2	Gram+/Gram−	[45]
Snakin-2	*Solanum tuberosum*	Snakins	66	7037.2	Gram+/Gram−	[46]
Vicilin-like Antimicrobial peptide 2a	*Macadamia integrifolia*	Vicilin-like	49	6103.6	Gram+	[47]
Vicilin-like Antimicrobial peptide 2b	*Macadamia integrifolia*	Vicilin-like	41	5110.6	Gram+	[47]
Vicilin-like Antimicrobial peptide 2c-1	*Macadamia integrifolia*	Vicilin-like	45	5905.5	Gram+	[47]
Vicilin-like Antimicrobial peptide 2c-2	*Macadamia integrifolia*	Vicilin-like	47	6189.9	Gram+	[47]
Vicilin-like Antimicrobial peptide 2c-3	*Macadamia integrifolia*	Vicilin-like	67	8864.7	Gram+	[47]
Vicilin-like Antimicrobial peptide 2d	*Macadamia integrifolia*	Vicilin-like	35	4642.1	Gram+	[47]
